# Solar-matched S-scheme ZnO/g-C_3_N_4_ for visible light-driven paracetamol degradation

**DOI:** 10.1038/s41598-024-60306-0

**Published:** 2024-05-28

**Authors:** Fahad Hassan, Sumina Namboorimadathil Backer, Ismail W. Almanassra, Muataz Ali Atieh, Mady Elbahri, Abdallah Shanableh

**Affiliations:** 1 Research Institute of Sciences and Engineering, University of Sharjah, Sharjah, 27272 UAE; 2 Chemical and Water Desalination Engineering Program, College of Engineering, University of Sharjah, Sharjah, 27272 UAE; 3 Nanochemistry and Nanoengineering, Department of Chemistry and Materials Science, School of Chemical Engineering, Aalto University, 02150 Espoo, Finland; 4 Department of Civil and Environmental Engineering, College of Engineering, University of Sharjah, Sharjah, 27272 UAE

**Keywords:** Zinc oxide, Graphitic carbon nitride, Photodegradation, Band alignment, Degradation pathway, Energy science and technology, Materials science, Nanoscience and technology

## Abstract

In pursuit of an efficient visible light driven photocatalyst for paracetamol degradation in wastewater, we have fabricated the ZnO/g-C_3_N_4_ S-Scheme photocatalysts and explored the optimal percentage to form a composite of graphitic carbon nitride (g-C_3_N_4_) with zinc oxide (ZnO) for enhanced performance. Our study aimed to address the urgent need for a catalyst capable of environmentally friendly degradation of paracetamol, a common pharmaceutical pollutant, using visible light conditions. Here, we tailored the band gap of a photocatalyst to match solar radiation as a transformative advancement in environmental catalysis. Notably, the optimized composite, containing 10 wt.% g-C_3_N_4_ with ZnO, demonstrated outstanding paracetamol degradation efficiency of 95% within a mere 60-min exposure to visible light. This marked enhancement represented a 2.24-fold increase in the reaction rate compared to lower wt. percentage composites (3 wt.% g-C_3_N_4_) and pristine g-C_3_N_4_. The exceptional photocatalytic activity of the optimized composite can be attributed to the band gap narrowing that closely matched the maximum solar radiation spectrum. This, coupled with efficient charge transfer mechanisms through S-scheme heterojunction formation and an abundance of active sites due to increased surface area and reduced particle size, contributed to the remarkable performance. Trapping experiments identified hydroxyl radicals as the primary reactive species responsible for paracetamol photoreduction. Furthermore, the synthesized ZnO/g-C_3_N_4_ composite exhibited exceptional photostability and reusability, underscoring its practical applicability. Thus, this research marks a significant stride towards the development of an effective and sustainable visible light photocatalyst for the removal of pharmaceutical contaminants from aquatic environments.

## Introduction

In recent years, pharmaceutical compounds present in wastewater have evolved paramount environmental concerns affecting aquatic and human life^1^. A plethora of drugs including antiseptics, antibiotics, disinfectants, and anti-inflammatory contents are being used extensively^2^. Above all, one of the most heavily consumed drugs is paracetamol, a well-known antipyretic and analgesic^3^. In connection to the enormous production and usage, paracetamol has been found commonly in environment, specifically in wastewater with 0.01–0.03 mg/L, which advocates the status of an unsafe drug^3^. The catastrophic effects of the contaminant could range from failure of many organs to unexpected death of humans and animals^3^. To eradicate the mentioned above, it is required to reduce the paracetamol into nontoxic products by an effective method. Many advanced processes of oxidation such as ultraviolet (UV) oxidation, ozonation^4^, electrochemical^5^ and heterogeneous photocatalysis^6^ have been utilized to curb the unwanted pharmaceutical compounds. Several metal oxide semiconductors having a higher band gap are formulated^7^ which includes titanium oxide (TiO_2_), ZnO and graphene-oriented metal oxides, these materials could disintegrate the organic pollutants by catalysis in UV light irradiation^8–10^. Moreover, a huge number of 2D S-scheme heterojunction photocatalyst systems have also been used for photocatalysis with various configurations of dimensions^11,12^.

Due to the immaculate mineralization activity for aqueous contaminants, destruction of organic-molecules, specific-organisms, and toxic metal ions, ZnO has been the most promising material^13,14^ which has executed its application in biomedical and environmental research. Although the unmatched redox potential and electron mobility has second the extensive use of ZnO as photocatalyst^15–17^, the low absorption in visible light, low photo absorption sensitivity and weak charge carrier separation have obstructed its practical applications. Therefore, the researchers have put their efforts to modify the stability and performance of ZnO photocatalyst by heteroatom-doping^18,19^, novel-metal composite^20,21^, surface functionalization with polymers such as polydopamine^22^ or coupling it with a semiconductor^10,23–25^. Furthermore, the fabrication of ZnO based S-scheme heterojunctions has evolved as an advanced solution to overcome the limitations attributed to the use of pristine ZnO in photocatalysis^12,26–28^. Gaowei Han et al., performed a study on inverse Opal ZnO@Polydopamine S-Scheme heterojunctions for H_2_O_2_ production, the unique formation of S-scheme impacted effective separation/transport of charge carriers, which resulted in the enhancement of overall photocatalytic properties of ZnO^22^. The formation of ZnO based S-scheme heterojunction is more feasible approach considering the superior performances in various photocatalytic reactions, it majorly utilized the synergistic effect of internal electric field and band bending, detailed study has been done on this advancement detailing the improvements in the photocatalysis^26^. Synergistic effect of IIEF and band bending in S-scheme heterojunctions overcomes the drawbacks of PC migration in TZS and ASSZH. Therefore, the S-scheme heterojunction achieves spatial separation of powerful PC, resulting in enhanced photocatalytic performance. Construction of ZnO-based S-scheme heterojunction provides a feasible approach to achieve efficient photocatalytic pollutant degradation.

Meanwhile, g-C_3_N_4_, a semiconductor having many advantages such as easy preparation from low cost and widely available precursors, narrow band gap, having no harmful metals, baring excellent thermal and chemical stability^29,30^, has been used by scientists for several applications^31,32^ which include, biosensors^33^, biochemical^34^ and photo/electrochemical applications^35^. An effective and novel way to modify the light absorbance with increased charge separation is by forming a nanocomposite by coupling ZnO and g-C_3_N_4_^36^. Ball milling and two step chemisorption method has been adopted in earlier studies^37,38^ to fabricate ZnO/g-C_3_N_4_ composite. Formation of g-C_3_N_4_ decorated ZnO composites were reported by Zhu et al.^39^ by calcination method. Further, vapor condensation was also used to coat the ZnO nanorods with g-C_3_N_4_^40^. For the optimum photocatalytic activity, the synthesis method plays a vital role in establishing a junction between g-C_3_N_4_ and ZnO^39^. Recently, flower like structure of g-C_3_N_4_ and ZnIn_2_S_4_ was reported which was functionalized by benzoic for hydrogen evolution with photocatalysis and to degrade the tetracycline hydrochloride, they used novel BiOCII/β-Bi_2_O_3_ composite under sunlight simulator^41^. Moreover, functional g-C_3_N_4_ has been formulated with a new design by co-polymerization^42^. Furthermore, noticeable progress in ZnO and g-C_3_N_4_ based S-scheme photocatalyst has been seen due to their unmatched electronic structure and stability. An enhancement of photocatalytic performance has been observed in S-scheme photocatalyst due to efficient charge transfer and separation which is highlighted in the recent studies in terms of pollutant degradation, CO_2_ reduction and water splitting^43^.

The significant efforts directed towards enhancing the efficiency of photocatalysts for visible light-driven processes of such composite, the utilization of g-C_3_N_4_ as a seed at very low concentrations, coupled with solid-state transformation techniques to tailor the morphology, surface porosity, and concentration while simultaneously engineering the band gap of ZnO to match the maximum solar absorption, represents an underexplored frontier in this domain. However, tailoring the band gap of a photocatalyst to match the solar radiation spectrum represents a pivotal advancement in the field of photocatalysis^26^ with profound implications for the degradation of contaminants.

Considering the above-mentioned traits we have fabricated a ZnO/g-C_3_N_4_ S-scheme photocatalyst and to the best of our knowledge, there has been a conspicuous absence of research addressing the crucial aspects mentioned above. Furthermore, the integration of such a structure for visible light-assisted catalysis of paracetamol, using a ZnO/g-C_3_N_4_ S-scheme photocatalytic composite, remains uncharted territory in the existing literature. Considering this, our study breaks new ground by introducing a novel approach. We present a meticulous synthesis of a composite material containing g-C_3_N_4_ and ZnO, achieved through a combination of hydrothermal and thermal oxidative methods with varying g-C_3_N_4_ ratios.

Our comprehensive investigation includes a thorough characterization of the synthesized composite, involving X-ray diffraction (XRD), Fourier transform infrared (FTIR) spectroscopy, field emission scanning electron microscopy (FESEM), energy dispersive X-ray spectroscopy (EDS), UV–Vis spectroscopy, Brunauer, Emmett, and Teller (BET) analysis, X-ray photoelectron spectroscopy (XPS), and liquid chromatography quadrupole time-of-flight mass spectrometry (LC-QTOF-MS). Through this analysis, we have systematically explored the structural, textural, and optical properties of our material.

The results of our study reveal the remarkable efficiency of this composite in the degradation of paracetamol in water under visible light irradiation. This enhanced performance can be attributed to several factors, including a reduction in the band gap, an increase in the specific surface area (S_BET_), and the effective transfer of charge carriers within the prepared photocatalytic composite. Notably, we have determined that a specific concentration of g-C_3_N_4_ in ZnO plays a pivotal role in the mineralization process. Additionally, we delve into the mechanism of band alignment through ultraviolet photoelectron spectroscopy (UPS) to provide insights into the charge transfer processes within the composite. This research represents a significant step forward in the quest for advanced photocatalysts tailored for visible light-driven applications, offering both a thorough characterization and mechanistic exploration of the synthesized composite material.

## Experimental

### Chemicals and reagents

Zinc chloride pure ≥ 99.9%, was obtained from Sisco Research laboratories, India. Melamine (C_3_H_6_N_6_, ≥ 99%) was bought from Sigma Aldrich, Germany. Sodium hydroxide (NaOH, 98%) pellets, and Methanol (CH_3_OH) were obtained from SD Fine-chem Limited, India. Paracetamol (C_8_H_9_NO_2_, Hema Pharmaceuticals, India, 99.98%) was selected as main pollutant for this study. As supplied, all analytical grade chemicals were utilized without performing any further treatments. Deionized (DI) water (0.055 µS cm^1^) was obtained using NANOpure Diamond by Barnstead, USA and was utilized throughout in the study.

### Synthesis of catalysts

*Synthesis of g-C*_*3*_*N*_*4*_ Melamine was utilized to synthesize g-C_3_N_4_ as reported earlier^44^. Briefly, melamine (15 g) contained in a semi closed ceramic-crucible was heated in the tube furnace at a temperature of 550 °C with heating rate of 2 °C/min for 4 h. The sample obtained by the reaction was let cool to 22 °C naturally and eventually the yellow g-C_3_N_4_ powder was collected. Which was further washed with DI water and then dried at 60 °C for 12 h and eventually grounded in mortar to obtain fine powder for composite preparation.

*Synthesis of ZnO* For ZnO preparation, firstly, a homogeneous solution ZnCl_2_ was prepared by solubilizing 13.6 g ZnCl_2_ in DI water (100 mL), labeled as solution A. Secondly, solution B was prepared by adding 3.9 g of NaOH in 50 mL of DI water at normal temperature. Later, solution A was heated to 35 °C with constant vigorous stirring while solution B was added to it in dropwise, precipitates of Zn(OH)_2_ were formed at this stage. The mixture was kept on stirring under same conditions for an hour to dissolve the precipitate completely and to get a homogeneous solution. The obtained homogeneous solution was then added to teflon lined reactor assembly for hydrothermal treatment at 120 °C for 24 h. After the treatment, the sample contained into a petri dish was left in the oven for drying at 60 °C for 12 h. The product was finally grounded in mortar to collect the fine powder of ZnO.

*Synthesis of ZnO/g-C*_*3*_*N*_*4*_* composite* To obtain a variety of g-C_3_N_4_ concentrations in composites, 0.03, 0.05, 0.1 and 0.2 g of already prepared g-C_3_N_4_ was added separately to 20 mL of methanol with 1 g of ZnO each to get ZnO/g-C_3_N_4_ composites of wt.% 3, 5,10 and 20 wt.%. The solution was kept at a constant stirring and heating at 50 °C till evaporation of methanol. The obtained product was then shifted to semi closed ceramic crucibles to heat at 500 °C for 2 h and 5 °C/min heating rate. The obtained composites were grounded in the mortar to obtain fine powder.

*Preparation of paracetamol stock* A 30 mg/L solution of paracetamol drug was prepared as initial concentration for the photodegradation analysis. An amount of 0.03 g paracetamol weighed accurately and added into 1000 mL of DI water with constant stirring for an hour to completely dissolve and form a homogenous stock solution.

### Characterizations

Powder XRD was performed using Bruker D8 advance and the diffraction patterns were collected between 10 and 80° of 2θ. XPS and UV-UPS was performed using XPS Nexsa G2, Thermo scientific, U.K with mono-chromatized Al- Kα radiation (1486.6 eV) under ultra-high vacuum (∼10 − 9 mbar), data was interpreted using the software Avantage V6.4.1. FTIR data was collected using Jasco 6300, the spectra were obtained in the range of wavenumber 500 – 4000 cm^−1^. FESEM was done for surface morphology, Tescan Vega 3XMU was utilized for the images and EDS. Specific surface area (S_BET_) was obtained using, NOVA tech Lx^2^ NT2LX-1, adsorption isotherm was analyzed on standard BET pressure range 0.05 < P/P_o_ < 0.3, employing nitrogen adsorbate with molecular area 16.2 Å^2^ at 77 K. UV–VIS absorbance spectra were investigated with Shimadzu UV-2600i, while the diffusive reflectance spectra were obtained by an integrated sphere attachment to the device.

### Photocatalytic Study

Photocatalytic performances studies of the fabricated catalysts were conducted under Xenon light source of 500 W power fitted with 420 nm UV cutoff filter. Briefly, the desired amount of the catalyst was added to a beaker containing 100 mL of paracetamol solution and placed at a distance of 5 cm under the light source. Initially, the solution was agitated in dark for 30 min to achieve adsorption desorption equilibrium. Later, visible light irradiation was employed on the solution for 60 min to initiate the photocatalytic process. At time intervals, 3 mL aliquots were taken for analysis by filtering through a 0.45 µm syringe-filter. The concentration of paracetamol was measured spectrophotometrically by absorbance in the solution at a wavelength of λ_max_ = 245 nm. Equation (1) was used to determine the percentage photodegradation of paracetamol, while the kinetic behavior of photodegradation was analyzed by fitting the investigated data with first-order kinetic model using Eq. (2).

1 $$Degradation \left(\%\right)=\frac{{C}_{0}-{C}_{t}}{{C}_{0}}\times 100\%$$

2 $${\text{ln}}\left(\frac{{C}_{t}}{{C}_{0}}\right)=-kt$$

Here, ${C}_{0}$ (mg/L) is absorbance of the solution after the adsorption process at 30 min in dark and ${C}_{t}$ (mg/L) is the absorbance of the pharmaceutical solution at time t, $k$ is the first-order rate constant.

The principal charge carriers accountable for degradation were elucidated by undertaking scavenging/trapping experiments. In our experiments, isopropanol (IPA, 10 mM), ammonium oxalate (AO, 10 mM) and p-benzoquinone (BQ, 10 mM) were added separately to the photocatalytic reaction conditions, these chemicals acted as scavengers for hydroxyl radical, hole and superoxide radical, respectively. For the reusability experiment, the solution was centrifuged after photocatalysis at 5000 rpm for five minutes, solid particles obtained by centrifugation were used again under same conditions for degradation. The pH of solution was altered between 4 and 11 to analyze the influence of pH on the photocatalytic effectiveness. The concentration of paracetamol was varied between 10 and 80 mg/L to identify the optimized value. Moreover, the amount of photocatalyst in degradation experiments was also changed between 10 and 25 mg with an increment of 5 mg in each experiment.

## Results and discussion

### Materials characterization

**Figure 1:**
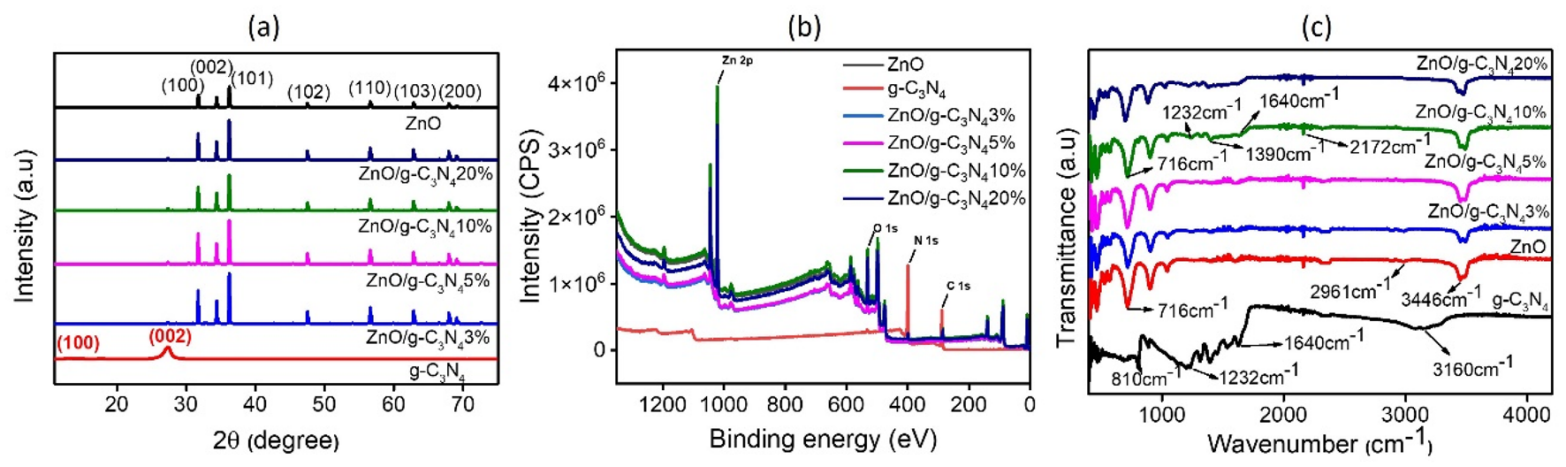
(**a**) XRD patterns of g-C_3_N_4_, ZnO and ZnO/g-C_3_N_4_ composites (**b**) survey scan of g-C_3_N_4_, ZnO and ZnO/g-C_3_N_4_ composites (**c**) FTIR spectra of g-C_3_N_4_, ZnO and ZnO/g-C_3_N_4_ composites.

The pure ZnO diffraction peaks as illustrated in Fig. 1a at 2θ of 31.7°, 34.5°, 36.3°, 47.6°, 56.7°, 62.8° and 68.1° correspond to (100), (002), (101), (102), (110), (103) and (200) planes respectively (JCPDS: 36-1451) validated the formation of wurtzite phase of ZnO^45^. For pristine g-C_3_N_4_, well-defined peaks at 12.8° and 27.3° were observed, which were referred to the diffraction planes (100) and (002) respectively (ICDD 00-050-1250)^46^. These peaks correspond to the interlayer and interplanar stacking of graphitic materials^47^. The smaller peaks at 12.8° corresponding (100) could be referred to in-plane structural arrangement of g-C_3_N_4_, for instance, h^+^ to h^+^ distance of nitride-pores^48^. Moreover, the diffraction patterns of all ZnO/g-C_3_N_4_ composites portray peaks matching ZnO along with a small peak at 27.3° corresponding to g-C_3_N_4_ suggesting the successful creation of composite between g-C_3_N_4_ and ZnO^49^. Furthermore, peak intensity ratio of g-C_3_N_4_ (002) to ZnO (101) is calculated for each composite, depicted in Table 1. For the ZnO/g-C_3_N_4_ composites (3, 5,10 and 20 wt.%), ratio was found to be increased which anticipated to the increased amount of g-C_3_N_4_.

**Table 1:** XPS surface elemental composition of synthesized material and XRD peak intensity ratio of g-C_3_N_4_ (002) to ZnO (101).

	ZnO	g-C_3_N_4_	ZnO/g-C_3_N_4_ 3%	ZnO/g-C_3_N_4_ 5%	ZnO/g-C_3_N_4_ 10%	ZnO/gC_3_N_4_ 20%
At%: Zn	35.51	–	39.99	40.22	38.86	34.61
At%: O	45.19	1.85	40.48	38.38	40.35	37.35
At%: C	–	42.43	15.64	16.83	17.97	20.26
At%: N	–	55.72	1.37	2.24	2.81	7.78
I_(002)_/I_(101)_	–	–	0.023	0.038	0.047	0.063

The XPS was performed to analyze the surface elemental composition of the composites. XPS survey scan presented in Fig. 1b confirms the presence of Zn, O, C and N elements, indicating the successful formation of composite between g-C_3_N_4_ and ZnO, where the pristine g-C_3_N_4_ only showed the presence of C and N elements as expected. Atomic percentage (At%) calculation of synthesized material was estimated from XPS survey spectra which is tabulated in Table 1. The increased At% of C and N in the composite was attributed to the increased loading of g-C_3_N_4_. For instance, it is clearly evident from the Table 1 that the atomic percentage of N in the composites was increased to 1.37, 2.24, 2.81 and 7.78 At% for ZnO/g-C_3_N_4_3%, ZnO/g-C_3_N_4_5%, ZnO/g-C_3_N_4_10% and ZnO/g-C_3_N_4_20%, respectively, ensuring the composition of g-C_3_N_4_ in the composites.

The structural information of the synthesized material in terms of functional groups was studied by FTIR and the outcomes are illustrated in Fig. 1c. The two peaks visible at wavenumber 1232 cm^−1^ and 1640, demonstrated in g-C_3_N_4_ spectrum, were attributed respectively to C–N and C=N stretching^50^. The evident peak corresponding to 810 cm^−1^ could be anticipated for the s-trizine ring breathing modes^51^. The broad absorption band in ZnO spectrum around 3000–3400 cm^−1^ was referred as the stretching vibration in −NH_2_ by N−H bonds and/or = N−H amines in addition to the hydroxyl groups which contains water molecules that are chemically and/or physically adsorbed^50,52^. The presence of main g-C_3_N_4_ characteristic peaks in ZnO/g-C_3_N_4_ suggested the appearance of structural features of g-C_3_N_4_ in the composite. Moreover, the increasing absorbance intensity was observed at 1232–1640 cm^−1^ as the amount of g-C_3_N_4_ increased^52^. Therefore, the FTIR analysis confirmed the occurrence of fundamental characteristic peaks pertaining to pure ZnO and pristine g-C_3_N_4_ in the composites.

**Figure 2:**
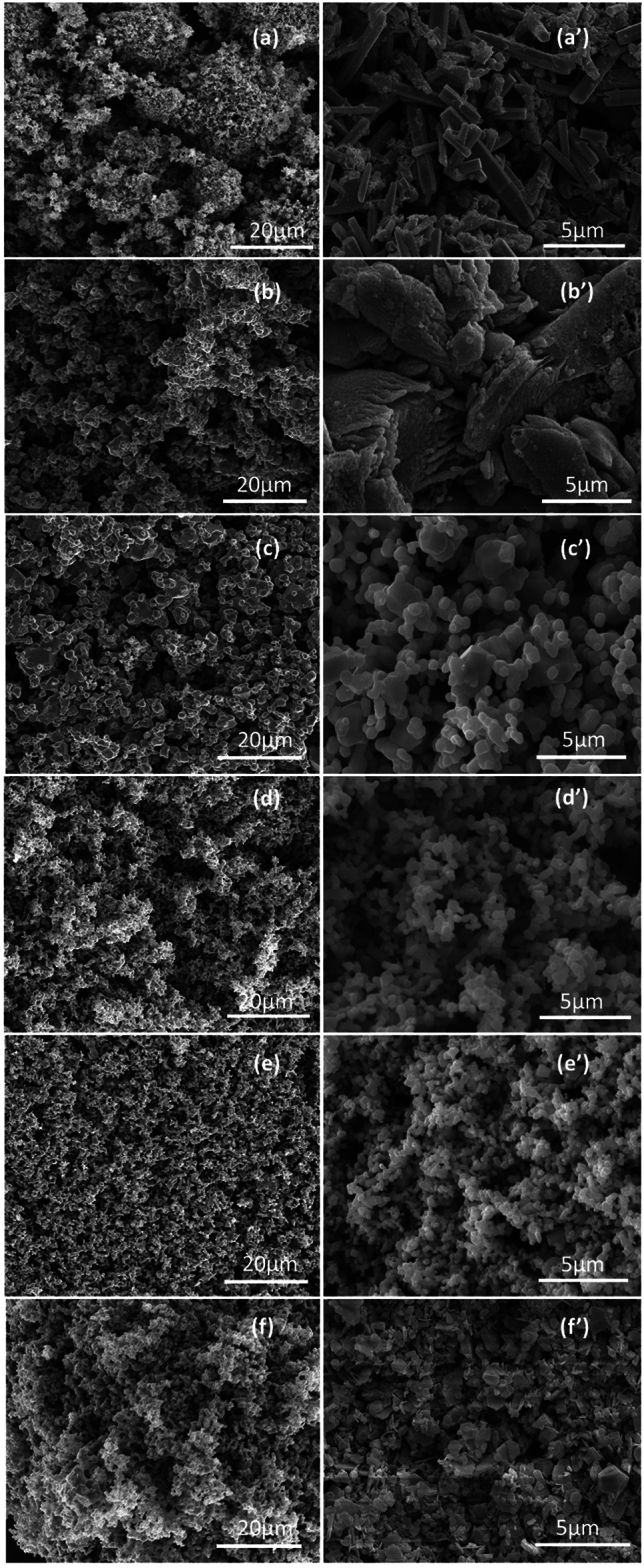
FESEM low and high magnification images of (**a**,**a’**) ZnO, (**b**,**b’**) g-C_3_N_4_, (**c**,**c’**) ZnO/g-C_3_N_4_3%, (**d**,**d’**) ZnO/g-C_3_N_4_5% (**e**,**e’**) ZnO/g-C_3_N_4_10% (**f**,**f’**) ZnO/g-C_3_N_4_20%.

Surface morphology of unblended ZnO, pristine g-C_3_N_4_ and their composites was investigated by FESEM. Figure 2a–f represented the large-scale morphology of synthesized materials. The high magnification FESEM image of ZnO (Fig. 2a’) possessed rod-like morphology^53^ having an average length of 2.77 µm, it has regular and almost uniform surface structure evidently demonstrated polyhexagonal crystal corresponds to the wurtzite phase of ZnO^45^. Figure 2b’ shows that g-C_3_N_4_ existed in thick dense aggregates with a mean size of 2.82 µm and a morphology resembling layered sheet as expected for graphitic materials^54^. The FESEM images (Fig. 2c’–f’) revealed that morphologies of ZnO/g-C_3_N_4_ composites are evidently unlike that of ZnO and g-C_3_N_4_ alone. As the mass loading of the g-C_3_N_4_ increased from 3 to 20 wt.%, coral like morphology was found to be formed^55^ which was different from the parent elements hence confirming the formation of composite as observed in the earlier studies^56^. Moreover, the uniform distribution and minimized aggregation in the composites maximized the reactive sites which could be favorable for photocatalytic reaction^57^. Unlike the ZnO and g-C_3_N_4,_ an obvious increase in roughness was noticed in the hybrids which could be ascribed to the uniform particle assembly on the surface during heating treatment^56^. Additionally, by the composite formation, the average particle size was greatly reduced from 2.82 µm to 384 nm.

**Figure 3:**
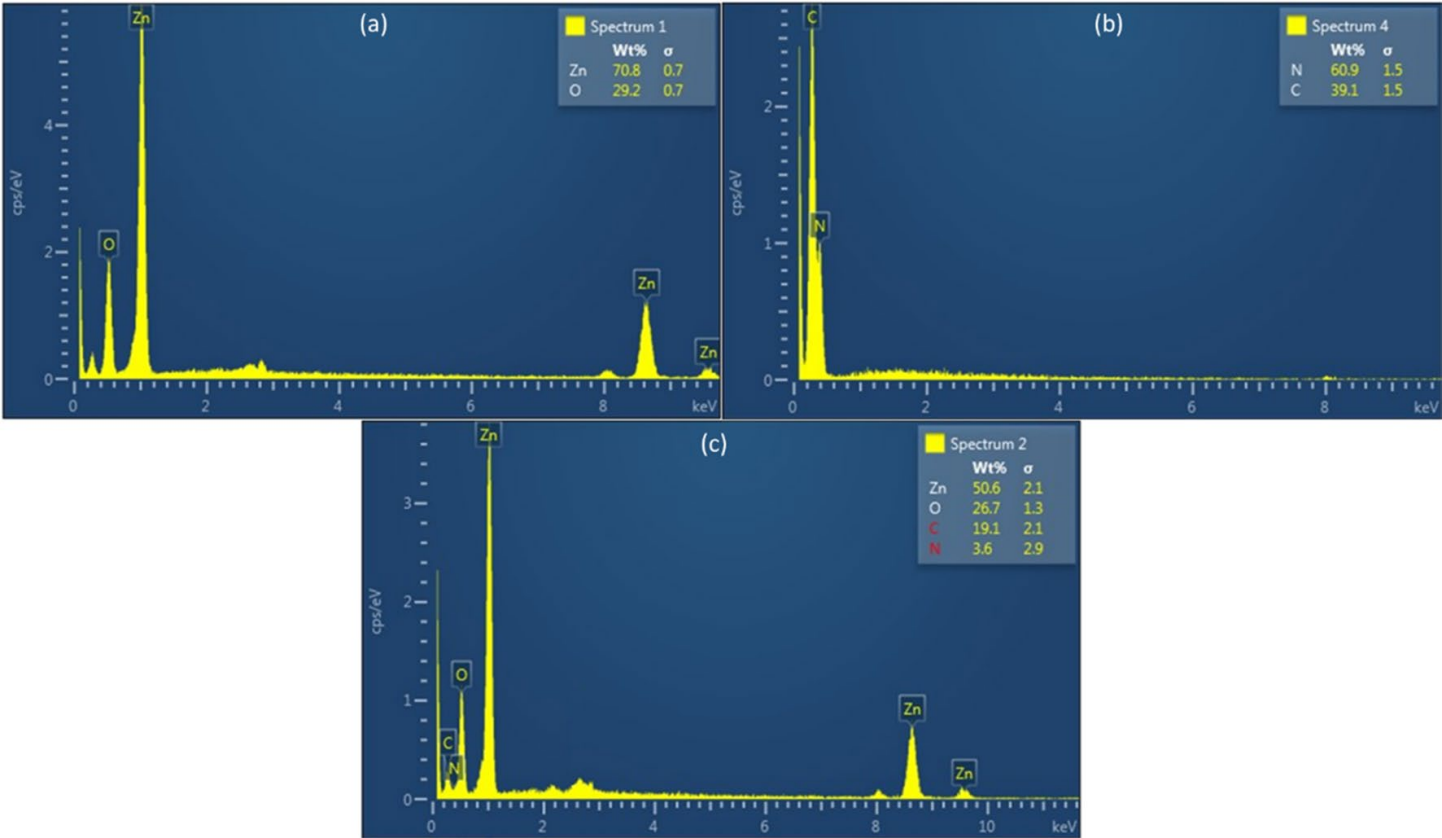
EDS of (**a**) ZnO, (**b**) g-C_3_N_4_ and (**c**) ZnO/g-C_3_N_4_10% composite.

The results providing the chemical compositions are demonstrated in Fig. 3. The EDS of pristine ZnO Fig. 3a depicts the existence of Zn and O, which indicates the phase pure formation. Similarly, the purity of prepared g-C_3_N_4_ was validated by the evident occurrence of C and N in Fig. 3b. Further, Fig. 3c shows the EDS of the optimized composite containing 10 wt.% g-C_3_N_4_ with ZnO which has provided the maximum degradation of paracetamol (it will be discussed in subsequent sections) and the existence of Zn, O, C and N elements without impurities justified the purity and accuracy of the synthesis procedure.

**Figure 4:**
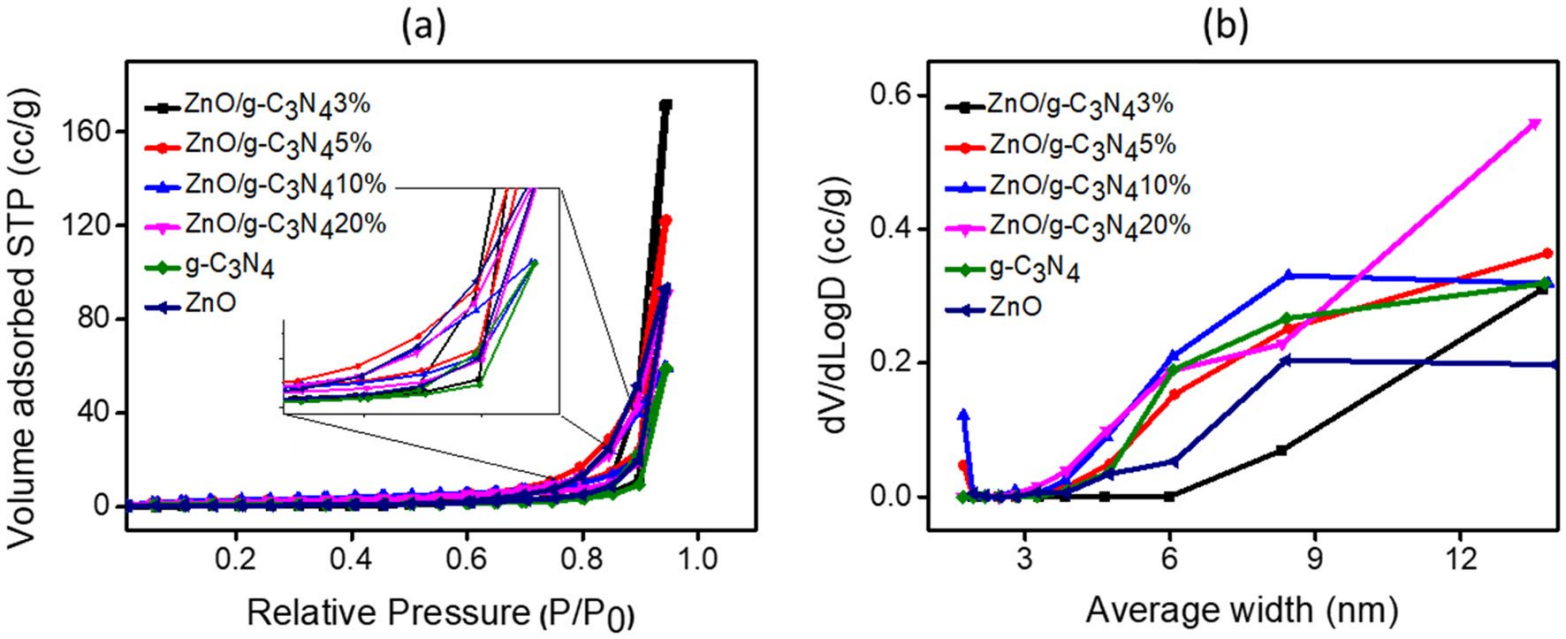
(**a**) Nitrogen adsorption and desorption isotherm with inset an enlarged view of hysteresis loop. (**b**) Pore size distribution of ZnO, g-C_3_N_4_ and the composites of ZnO/g-C_3_N_4_.

To determine the catalytic activity of the nanomaterials, S_BET_ is an important parameter to be taken into view. A higher S_BET_ is accountable for enhanced adsorption of molecules and reduced reaction time. Figure 4a. illustrates nitrogen adsorption/desorption isotherms for pure ZnO, g-C_3_N_4_ and their composites. The isotherms of adsorption were conducted at 77.3 K at 0.01 to 0.95 P/P_0_ relative pressure. The findings revealed that all the composites have a similar isotherm classified as classical type V isotherm hysteresis loop in accordance with IUPAC classification^58^, indicating that all the surfaces of composites were mesoporous. In the relative pressure region from 0.6 to 0.9 P/P_0_ (see inset of Fig. 4a), the hysteresis loop belongs to H3 type^59^, implying the presence of parallel plate slit pore structure^60^. The data for S_BET_ of ZnO, g-C_3_N_4_ and the composites is provided in Table 2. Interestingly, the surface area measurements followed the surface morphology of the composites (see Fig. 2). As seen from the monographs, the composites look well distributed by raising the g-C_3_N_4_ loading from 3 to 10 wt.%, this distribution was even more evident at 10 wt.% explaining the increase in the S_BET_ by increasing the wt.% of g-C_3_N_4_. This factor was related to the high N_2_ volume adsorption at higher relative pressure as a result of capillary condensation in mesopores^55^. However, as the loading of g-C_3_N_4_ increased further to 20 wt.%, the sheets of the g-C_3_N_4_ looked dominated and covered the ZnO particles (see Fig. 2) revealing a decrease in the S_BET_ which could also be devoted to the blockage of partial g-C_3_N_4_ nano-sheets^61^. Further, pore size and pore volume distribution was also determined. The average pore diameter ranged between 2 and 4 nm as displayed in Fig. 4b. Improved S_BET_ for the optimized composite could be beneficial for the photocatalytic studies as it is responsible for redox reaction by increasing active sites^62^.

**Table 2:** Kinetic rate constant, summary of S_BET_, band gap energies and relative % degradation by photocatalysts.

Catalyst	R^2^	Rate constant × 10^–2^ (min^−1^)	S_BET_ (m^2^/g)	Band gap energy (eV)	Degradation (%)
ZnO	–	–	2.335	3.13	4
g-C_3_N_4_	0.997	2.34	2.728	2.78	75
ZnO/g-C_3_N_4_3%	0.987	2.6	4.091	2.75	78
ZnO/g-C_3_N_4_5%	0.997	3.411	9.935	2.51	87
ZnO/g-C_3_N_4_10%	0.998	5.242	11.335	2.39	95
ZnO/g-C_3_N_4_20%	0.996	2.934	8.137	2.67	82

**Figure 5:**
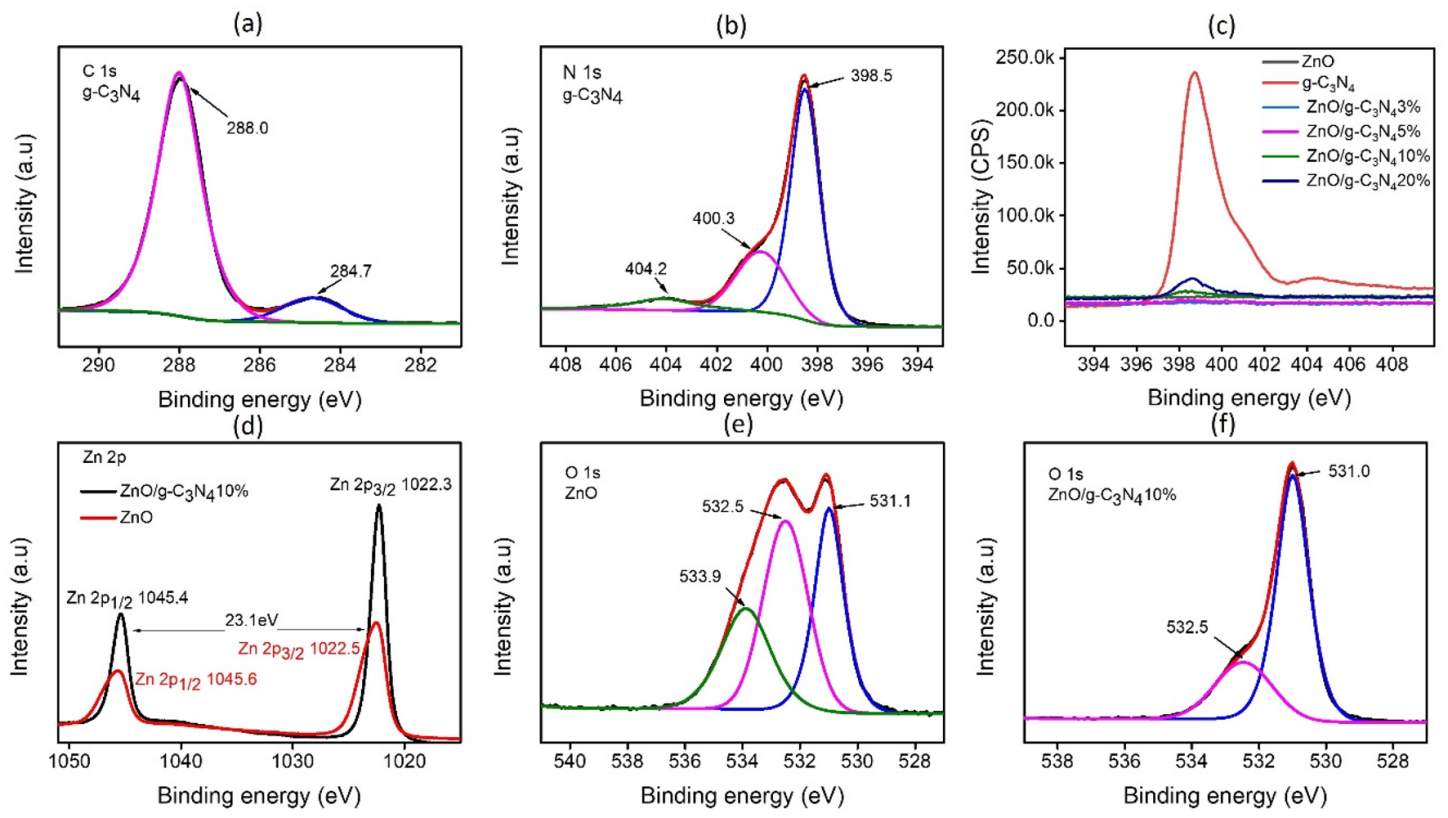
(**a**) high resolution C 1s spectra of g-C_3_N_4_ (**b**) high resolution N 1s spectra of g-C_3_N_4_ (**c**) high resolution N 1s spectra of g-C_3_N_4_, ZnO and ZnO/g-C_3_N_4_ composites (**d**) high resolution Zn 2p spectra of ZnO/g-C_3_N_4_10% and ZnO (**e**) high resolution O 1s spectra of ZnO (**f**) high resolution O 1s spectra of ZnO/g-C_3_N_4_10%.

The surface chemical composition of the formed ZnO, g-C_3_N_4_ and ZnO/g-C_3_N_4_10% composite was further examined using XPS (Fig. 5). The C 1s spectrum of g-C_3_N_4_ was resolved into two component peaks at 284.7 eV and 288.0 eV (Fig. 5a). The one at 284.7 eV was assigned to the aromatic C atom in the carbon nitride matrix (C−C bond) whereas, the other at 288.0 eV was designated to the sp2-bonded carbon attached to the NH_2_ group N=C–N^39,63,64^. The N 1s spectrum contained two major peaks along with a lower peak (Fig. 5b), the first at 398.5 eV was attributed to the aromatic nitrogen bonded to two carbon atoms (C=N−C), indicating the appearance of triazine rings^65^. The second shoulder peak at 400.3 eV was linked to the tertiary N atoms ((C−N(−C)−C or C−N(H)−C))^66^ while the last peak at 404.2 eV was referred to the charging impacts^67^. The high resolution N1s spectra of synthesized materials (Fig. 5c) showed the increased intensity, indicating the increased content of g-C_3_N_4_ in composite from 3 to 20 wt%. Provided in the Zn 2p spectrum of ZnO/g-C_3_N_4_10% composite, two major peaks at binding energies of 1045.4 eV and 1022.3 eV were assigned to Zn 2p_1/2_ and 2p_3/2_ lines respectively (Fig. 5d). The 23.1 eV binding energy separation between these two peaks indicated + 2 states of Zn ions in the composite^39^. However, a shift towards lower binding energy in the composite occurred, indicating the higher electron density in ZnO upon the formation of composite. It means that on contact there is charge transfer from g-C_3_N_4_ to ZnO^27^. The deconvoluted O 1s line (Fig. 5e) exhibits components at binding energy 531.1 eV, 532.5 eV and 533.9 eV respectively. The first can be linked to the existence of ZnO which is reduced partially such as, ZnO_x_, that is in close relation to the presence of O_2_^−^ ions generated by deficiencies of oxygen in the matrix of ZnO^68^. While the second component could be related to physically or chemically adsorbed O_2_, OH, and H_2_O on the surface^69^. The 533.9 eV peak refers to chemisorbed oxygen or surface hydroxyl bonding of Zn-OH^70^. The 531.0 eV peak in the O 1s spectrum (Fig. 5f) was linked to the participation of O^2−^ particles in zinc and oxygen bonding within the wurtzite ZnO structure^39^. Furthermore, the peak positioned at 532.5 eV could be related to the adsorbed hydroxide group displayed on the surface of composite^39^. In conclusion, the sturdy interfacial bonding encouraged shift of the absorption edge towards the visible region, which could bolster the photocatalytic efficiency by utilizing the maximum spectrum of solar energy^39,66^.

**Figure 6:**
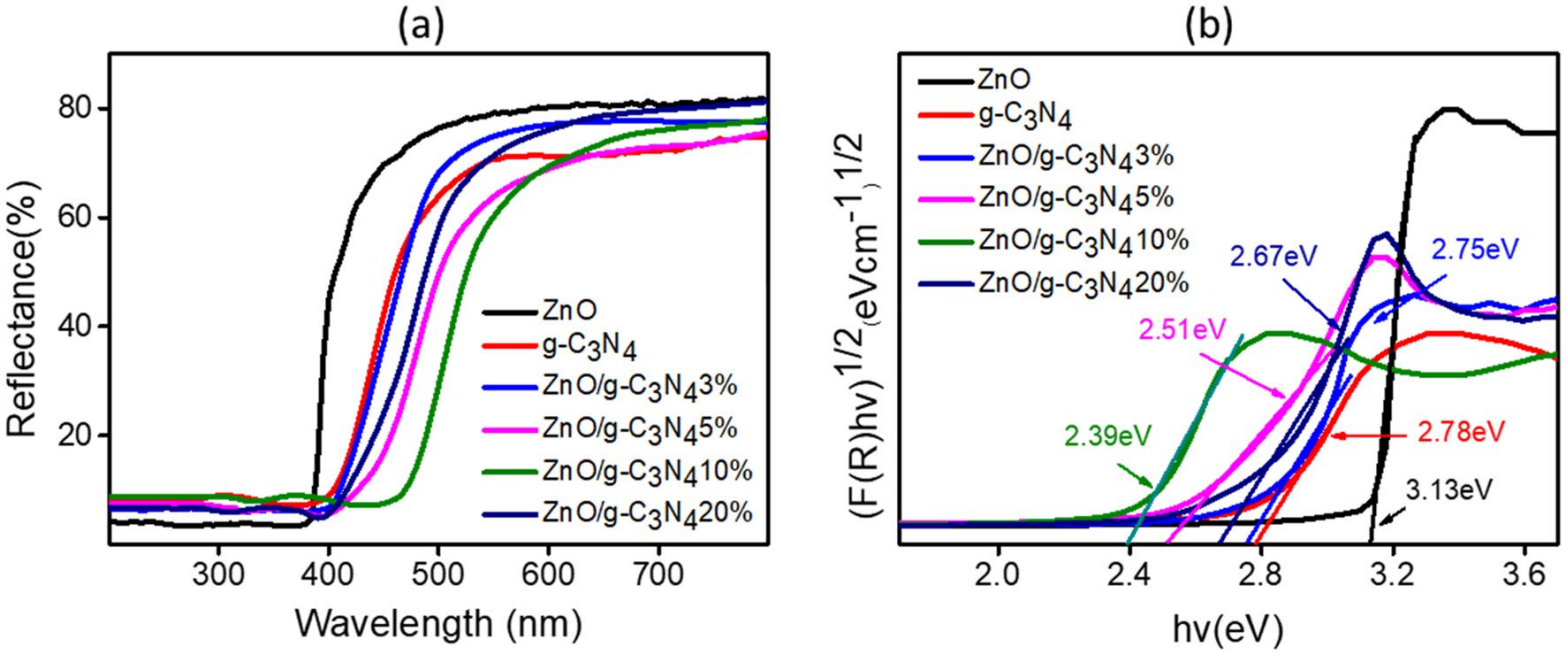
(**a**) UV–Vis diffusive reflection spectra and (**b**) K-M plot of ZnO, g-C_3_N_4_ and different compositions of ZnO/g-C_3_N_4_.

Optical properties illustrated in Fig. 6a represent the results obtained by UV–vis DRS spectra. The absorption band of ZnO (wavelength < 400 nm) was observed as earlier reported^71^, while the absorption band edge of composites in correlation to g-C_3_N_4_ and ZnO, experienced a red shift towards higher wavelengths extending between 400 and 460 nm. By the increment in g-C_3_N_4_ loading, the absorption range of composites was also promoted which resulted in improved utilization of solar light^61^. The band gap energies of g-C_3_N_4_, ZnO and ZnO/g-C_3_N_4_ composites were evaluated from Kubelka–Munk (KM) function and determined by slope of the line part to (ℎν) on the horizontal-axis as displayed in Fig. 6b. From the results shown in Table 2, a decline in band gap with increase in g-C_3_N_4_ wt.% is evident. Provided a band gap value of 2.39 eV for the optimized composite ZnO/g-C_3_N_4_10%, that match the maximum solar absorption spectrum and hence indicates that the optimized photocatalyst is an efficient candidate to initiate the visible light activated photocatalysis. An effective interaction of g-C_3_N_4_ and ZnO implied to a well bonded interface among ZnO and g-C_3_N_4_ phase while forming the composite, hence lowered the band gap energies^51,72^. Thereby, it can provide chances for delayed recombination and extensive transfer of charge carriers^73^. However, a slight increment in the band gap energy on further increasing the g-C_3_N_4_ loading to 20% could be linked to the quantum size of the active nano particles, electronic interphase impacts and/or smaller interaction between the phases as reported in earlier studies^55^.

### Photocatalytic study of paracetamol using ZnO/g-C_3_N_4_ composite

**Figure 7:**
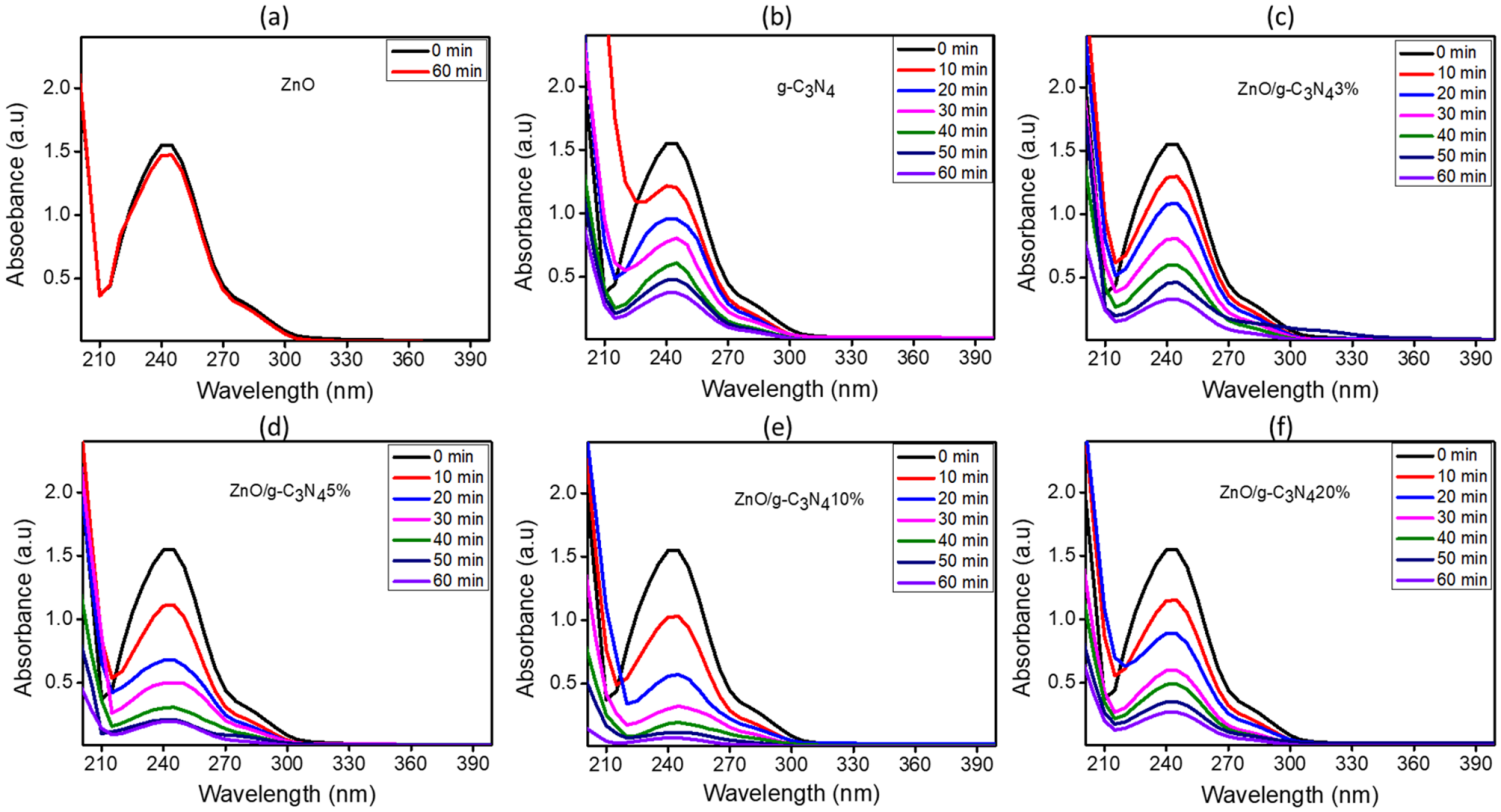
UV–Vis absorption spectra for ZnO, g-C_3_N_4_ and different compositions of ZnO/g-C_3_N_4_.

The results obtained by executing UV–Vis spectroscopy measurements ranging from 200 to 400 nm after photodegradation study of paracetamol are shown in Fig. 7a–f. It is evident from the results that unification of ZnO and g-C_3_N_4_ led to higher degradation of the pharmaceutical under study. It can be observed that paracetamol degradation of only 4% took place by utilizing pure ZnO (Fig. 7a), due to this negligible activity ZnO was not utilized in further experiments. However, the ZnO/g-C_3_N_4_10% as shown in Fig. 7e exhibited enhanced photoactivity and evidently degraded (95%) of the paracetamol in aqueous solution. The amplification in photocatalysis could be because of the separation of charge-carriers, enhanced morphology, lower band gap and higher S_BET_.

**Figure 8:**
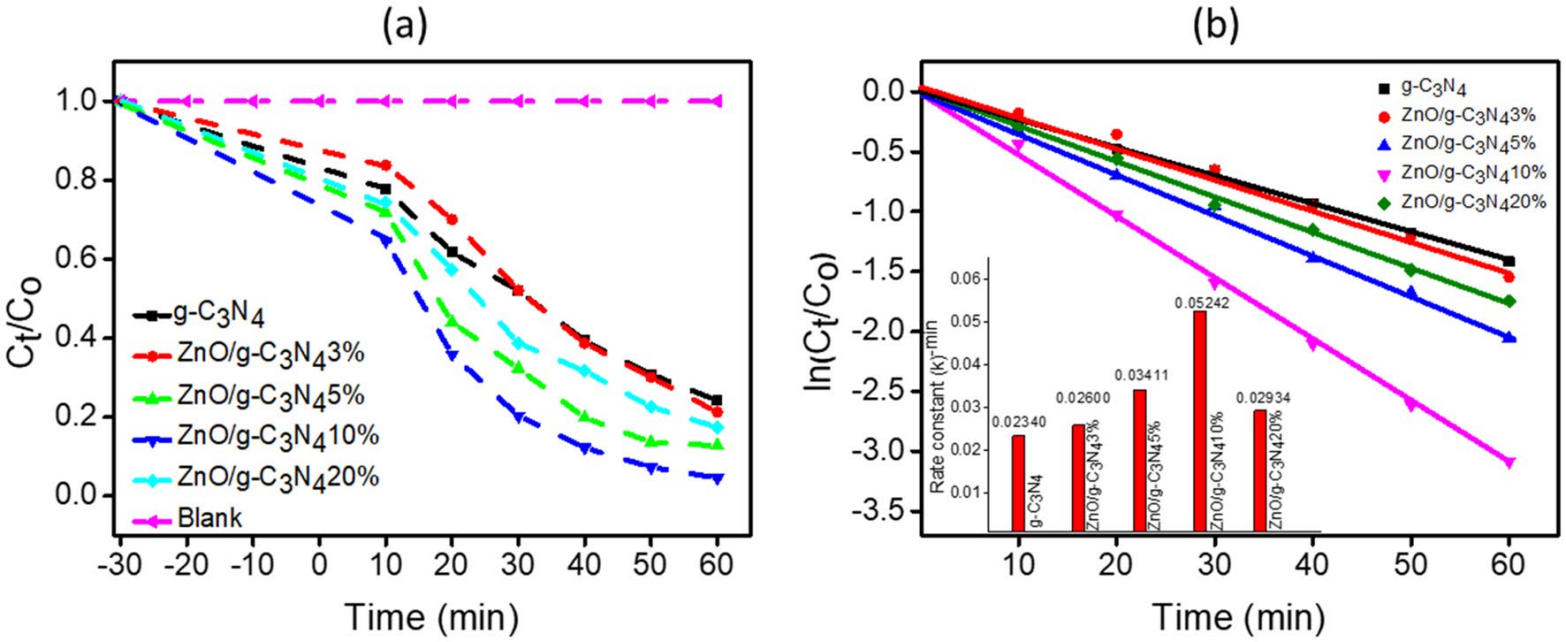
(**a**) Photodegradation of paracetamol versus time (**b**) ln (C_t_/C_0_) versus time and inset rate constant.

The photocatalytic performance results of fabricated catalysts are demonstrated in Fig. 8a. In 60 min of visible light irradiation, 75% of paracetamol was photocatalytically degraded by pure g-C_3_N_4_, while 78, 87, 95 and 82% degradation was observed by using ZnO/g-C_3_N_4_3%, ZnO/g-C_3_N_4_5%, ZnO/g-C_3_N_4_10%, and ZnO/g-C_3_N_4_20% respectively under same reaction conditions. Thus, the maximum photodegradation of paracetamol was performed by ZnO/g-C_3_N_4_10% composite. The order of photocatalytic performance was concluded as, ZnO/g-C_3_N_4_10% > ZnO/g-C_3_N_4_5% > ZnO/g-C_3_N_4_20% > ZnO/g-C_3_N_4_3% > g-C_3_N_4_.

Based on experimental results, a fitting to Eq. (2) was employed for the investigation of kinetic performance of photodegradation and the kinetic plots, ln (C_t_/C_0_), versus illumination time are exploited in Fig. 8b. From the kinetics outcomes it is obvious that the experimental data is well aligned with pseudo-first-order kinetics and fits well with the kinetic model. Table 2. contains a list of rate-constant (k) along with the correlation-coefficient (R^2^). From the data, the highest value of k was determined 5.242 × 10^–2^ min^–1^ for the ZnO/g-C_3_N_4_10% photocatalyst, that is much greater compared to pristine g-C_3_N_4_ (2.340 × 10^–2^ min^–1^) for the paracetamol degradation under similar reaction conditions.

In conclusion, by increasing g-C_3_N_4_ wt.% up to a certain amount, a boost in catalytic performance was observed due to effective charge carrier transportation between g-C_3_N_4_ to ZnO and suppressed rate of recombination of e^−^/ h^+^ pair^74^. However, a performance reduction was observed for the composite with more wt.% of g-C_3_N_4_ than the optimized value due to trapping and poor transfer of electrons followed by increased band gap^53^. After optimizing the suitable photocatalyst i.e., ZnO/g-C_3_N_4_10%, different parameters such as the influence of catalyst amount, drug dosage and pH were also examined.

### Effect of parameter on photocatalytic study

**Figure 9:**
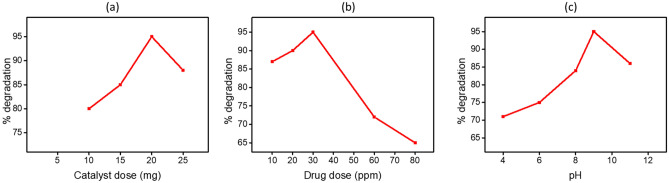
(**a**) Effect of initial catalyst (ZnO/g-C_3_N_4_10%) dose on the photodegradation of paracetamol (**b**) Influence of initial drug dose on the photodegradation of paracetamol using (ZnO/g-C_3_N_4_10%) (**c**) Impact of pH on the photodegradation of paracetamol using (ZnO/g-C_3_N_4_10%).

The results obtained by monitoring the influence of changing different factors and their impact on the efficiency of photodegradation are depicted in Fig. 9. The impact of photocatalyst quantity in solution and consequent impact on the degradation performance of paracetamol was studied by manipulating the amount of optimized photocatalyst, ZnO/g-C_3_N_4_10%, between 10 and 25 mg in 100 mL drug solution, the outcomes are provided in Fig. 9a. Initially, an increasing trend in degradation efficiency between 80 to 95% was observed by increasing the photocatalyst dose between 10 to 20 mg. However, a drop of efficiency from 95 to 88% was observed by further increasing the catalyst dose amount to 25 mg. For catalyst dosage < 20 mg, the lower photoactivity can be correlated to the limited number of active-sites for redox reactions. Whereas, the enhancement of photoactivity for dosage 20 mg was attributed to the availability of active sites^75^. However, a drop in effective degradation on furthermore addition of catalyst was credited to the reduced surface area because of the aggregation of catalyst composite resulting in low absorption capacity of photons^49^. Moreover, increased concentration could lead to more scattering of light photons, thereby resulted in poor penetration^53^. Hence, 20 mg dosage of optimized catalyst was selected as the ideal amount for photoactivity.

Another essential parameter in photocatalytic process is the initial drug concentration. To investigate the optimal value of paracetamol concentration, experiments were conducted by changing the paracetamol concentrations between 10 to 80 mg/L and utilizing 20 mg of optimized catalyst. The resultant photodegradation efficiencies were calculated as 87%, 90%, 95%, 72% and 65% for the 10, 20, 30, 60 and 80 mg/L of paracetamol concentration respectively (Fig. 9b). Provided the maximum degradation efficiency, 30 mg/L concentration of paracetamol was selected as an optimal value. At initial pollutant concentration lower than 30 mg/L, the reason for reduced efficiency could be that a trivial quantity of paracetamol was adsorbed on photocatalyst^76^. While, in concentration greater than 30 mg/L, the decrease in efficiency could be reasoned by suppressed path-length and lower penetration of photons^77^.

The photocatalytic performance was observed to be highly influenced by varying the solution’s pH between 4 and 11 while fixing the photocatalyst dose to 20 mg in 100 mL solution containing 30 mg/L paracetamol. Results provided in Fig. 9c show that at pH 4, 71% paracetamol was degraded followed by 75% degradation on increasing pH value to 6. The increasing trend in degradation efficiency sustained and was increased considerably providing a maximum degradation performance (95%) at pH 9. However, a drop in photodegradation efficiency was observed in stronger alkaline medium at pH 11. At low pH, the consequent lower degradation efficiency could be explained as excessive adsorption of H^+^ ions initiated a positive charge on the surface of photocatalyst which caused an electrostatic repulsion to the positive molecules of paracetamol. In comparison, a higher degradation efficiency in alkaline solution of pH 7 and above could be due to a negative charge acquired by the catalyst surface by adsorption of OH^−^ which then electrostatically attracted the positively charged paracetamol molecules^78^. Moreover, the degradation rate could be triggered by the creation of hydroxyl (•OH) radicals by OH^−^ in alkaline solution. However, a drop in degradation performance in stronger alkaline solution could be a result of struggle between OH^−^ and paracetamol molecule to be adsorb on catalyst surface^53^.

### Reusability test, TOC removal and charge carrier trapping/scavenging analysis

**Figure 10:**
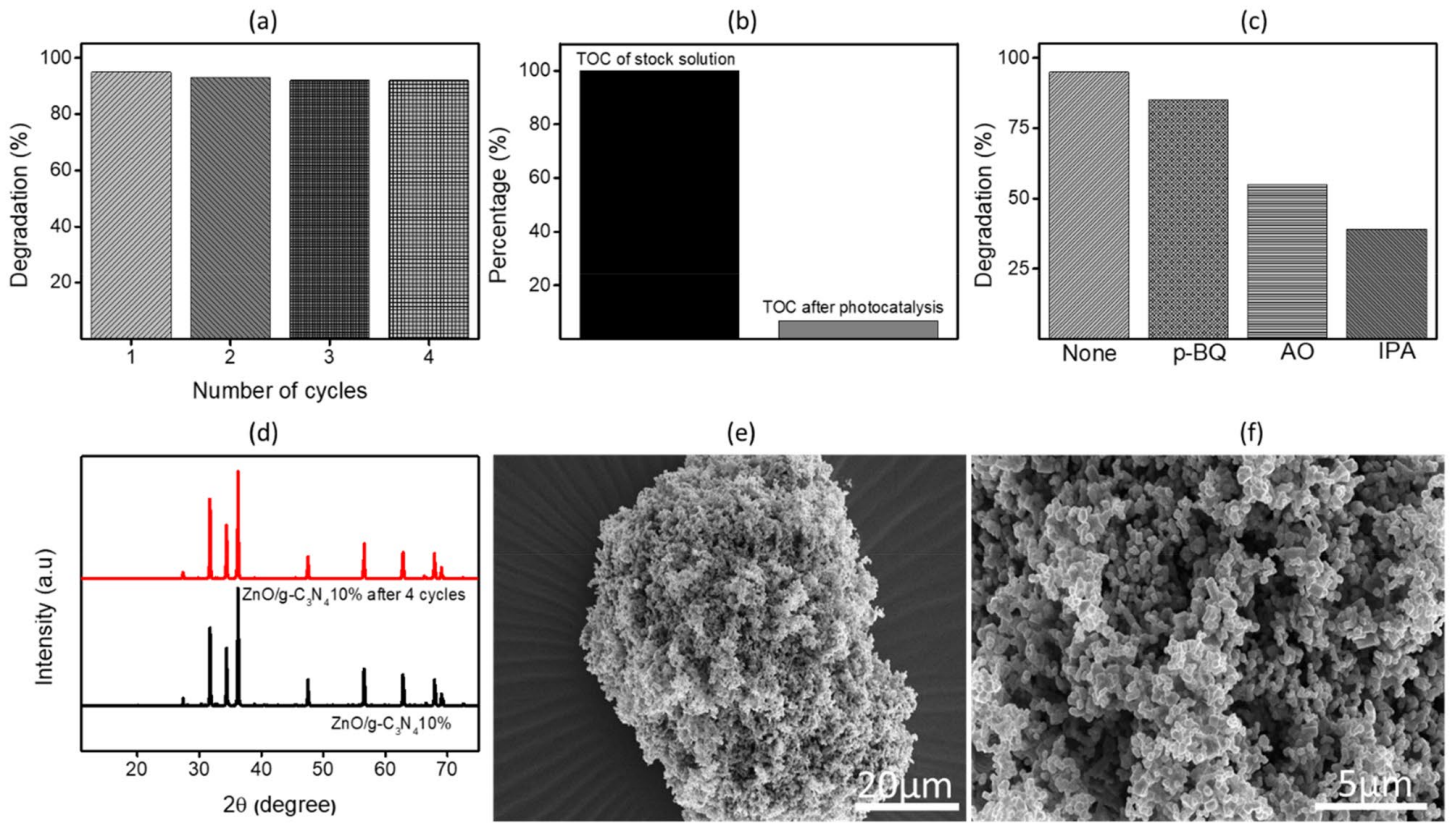
(**a**) Recyclability of ZnO/g-C_3_N_4_10% catalyst in 4-run of cycles (**b**) Percentage of TOC before and after degradation of paracetamol (**c**) charge carrier trapping/scavenging analysis (**d**) XRD of ZnO/g-C_3_N_4_10% before and after 4 cycles (**e**–**f**) FESEM images of ZnO/g-C_3_N_4_10% after 4 cycles.

The stability and reusability performance of ZnO/g-C_3_N_4_10% photocatalyst was determined by repetitive four cycles under the same experimental conditions. After each cycle, the photocatalytic composite was recollected and centrifuged at 5000 rpm for 5 min followed by three time washing with distilled water. Before utilizing with a fresh paracetamol solution, the composite obtained after washing was dried in oven at 70 °C till all the water evaporated. As illustrated in Fig. 10a the optimized photocatalyst showed almost consistent photocatalytic efficiency over the four successive cycles. Repetitive runs showed a slight decrement of efficiency (only 3%) between four cycles which revealed the high stability of prepared photocatalyst under visible light illumination and confirmed the reusability several times. Since photocatalyst forms stable suspension with water, there are some chances of losing the sample which could also account for the faint drop in the photoactivity. However, overall study revealed the consistency in percentage degradation of paracetamol in multiple cycles which indicates the promising wide applicability of ZnO/g-C_3_N_4_10% photocatalyst.

To evaluate the extent of mineralization of paracetamol, total organic carbon (TOC) measurement was done for the irradiated solution. Initially the TOC value of stock solution before photocatalysis was 27.4 ppm whereas, after photocatalysis it reduced to 1.88 ppm. Figure 10b shows that 93% of TOC removal was achieved when 95% degradation of paracetamol by ZnO/g-C_3_N_4_10% composite occurred. The findings indicate that the mineralization of paracetamol is consistent with degradation.

The expected reactive species taking part in the photocatalytic study are commonly holes (h^+^), hydroxyl and superoxide radical (O2^•−^). To authenticate the capacity of reactive species involved in photocatalysis of paracetamol, charge carrier trapping/scavenging analysis was performed. Isopropanol (IPA) as (^•^OH), ammonium oxalate (AO) as (h^+^) and p-benzoquinone (BQ) as (O2^•−^) scavengers were used. Scavengers were added separately before the start of each experiment with 10 mM concentration in 100 mL. The data presented in Fig. 10c, determines a significant drop in degradation percentage from 95 to 39% upon the addition of IPA which confirms the dominance of (^•^OH) radicals and their accountability for the photodegradation of paracetamol. To prove the structural stability of photocatalyst, the XRD and FESEM analysis of spent ZnO/g-C_3_N_4_10% was analyzed after four cyclic usages. Figure 10d demonstrates stability by showing no visible change in the XRD pattern of ZnO/g-C_3_N_4_10% photocatalyst before and after the usage. Moreover, FESEM images (Fig. 10e–f) of spent ZnO/g-C_3_N_4_10% clearly depict no change in the morphology compared with surface morphology before usage (see Fig. 2e,e’). Hence, it is confirmed overall stability of ZnO/g-C_3_N_4_10%, suggesting that there is no deterioration of active component during photocatalysis.

### Band alignment and photocatalytic degradation mechanisms

**Figure 11:**
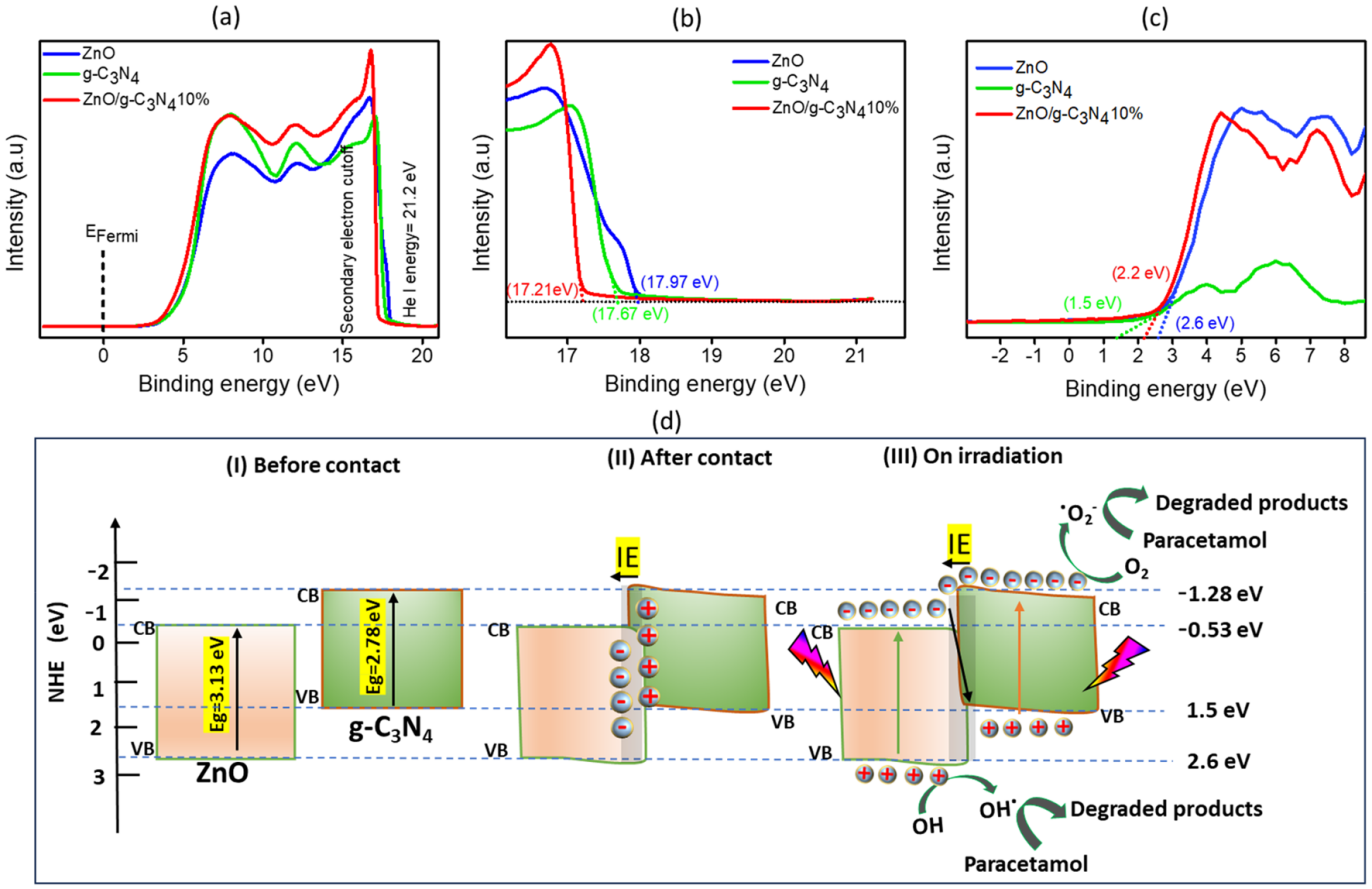
(**a**) UPS spectra, (**b**) He I spectra of secondary electron cutoff, (**c**) valence band maximum spectra of ZnO, g-C_3_N_4_ and ZnO/g-C_3_N_4_ (**d**) S-scheme mechanism of photodegradation of paracetamol using ZnO/g-C_3_N_4_10% photocatalyst.

To explore the mechanism of charge carrier transport in ZnO/g-C_3_N_4_10% composite, as well as in pure ZnO and pure g-C_3_N_4_, the ultraviolet photoelectron spectroscopy (UPS) was employed with He I excitation energy ($$h\vartheta$$ = 21.2 eV), and the outcomes were depicted in Fig. 11a. The analysis of the UPS spectra changes in the composite involved studying the secondary electron energy cutoff ($${E}_{cutOff}$$) and the valence band maximum (VBM). This was achieved by extrapolating the linear fit to the binding energy cutoff and identifying the intersection point with the UPS spectra baseline^79^. The determined ($${E}_{cutOff}$$) values were 17.97 eV, 17.67 eV, and 17.21 eV for ZnO, g-C_3_N_4_, and ZnO/g-C_3_N_4_10%, respectively (shown in Fig. 11b). Additionally, the VBM values were estimated as 2.6 eV, 1.5 eV, and 2.2 eV with respect to ZnO, g-C_3_N_4_, and the optimized composite (displayed in Fig. 11c). Using the ($${E}_{cutOff}$$) and VBM values, the energy of the valence band ($${E}_{VB})$$ and conduction band ($${E}_{CB}$$) for ZnO, g-C_3_N_4_, and ZnO/ g-C_3_N_4_10% was calculated through Eqs. (3) and (4)^80,81^. $${E}_{CB}$$ of g-C_3_N_4_ (− 1.28) was more electronegative than that of ZnO $$({E}_{CB}$$= − 0.53).

3 $${E}_{VB}=h\vartheta -\left|{E}_{cutOff}-VBM\right|$$

4 $${E}_{CB}={E}_{v}-{E}_{g}$$

The schematic in Fig. 11d depicts the mechanism of charge transfer for photocatalytic degradation by the optimized catalyst which is found to be following S-scheme mechanism^12,26–28^. It can be explained as, before contact (Fig. 11dI), ZnO and g-C_3_N_4_ have separate band structure and photogenerated electrons could form upon the absorption of energy suitable to band gap. After the contact (Fig. 11dII), electrons would freely transfer from g-C_3_N_4_ to ZnO, till the fermi lever reaches the equilibrium. At that moment ZnO befits more negative charge, whereas g-C_3_N_4_ becomes more positive, which created an internal electric field (IE) at the interface. It resulted in band edge bending in both systems. On irradiation (Fig. 11dIII), naturally ZnO and g-C_3_N_4_ got excited from VB to CB creating the electron hole pair. In the meantime, IE field at the interface and suitable band bending attributed to the recombination of photogenerated electron in ZnO with photogenerated holes in g-C_3_N_4_ which subsequently facilitated the preservation of photogenerated electron in g-C_3_N_4_ and photogenerated holes in ZnO. The resulted efficient separation and delayed recombination paid the enhanced redox activity for the optimized composite. The photogenerated electron in CB of g-C_3_N_4_ reacted with dissolved molecular oxygen forming superoxide anion i.e. O^2^•^−^ which inducted the paracetamol degradation^50^. In parallel reaction, photo-excited holes on the VB of ZnO interacted with the water molecule and got reduced to form hydroxyl radicals (^.^OH)^57^. As a result of redox reaction, almost complete photodegradation of paracetamol was achieved. The photocatalytic reactions that occurred are shown in Eqs. (5–8) below.

5 $${\text{O}}_{{2}} + {\text{ CB}}^{{{\text{e}} - }} \to^{ \bullet } {\text{O}}_{{2}}^{ - }$$

6 $$^{ \bullet } {\text{O}}_{{2}}^{ - } + {\text{ paracetamol }} \to {\text{ degradation products}}$$

7 $${\text{VB}}^{{{\text{h}} + }} + {\text{ H}}_{{2}} {\text{O}} \to^{ \bullet } {\text{OH}}$$

8 $$^{ \bullet } {\text{OH }} + {\text{ paracetamol }} \to {\text{degradation products}}$$

### Possible photodegradation pathway

**Figure 12:**
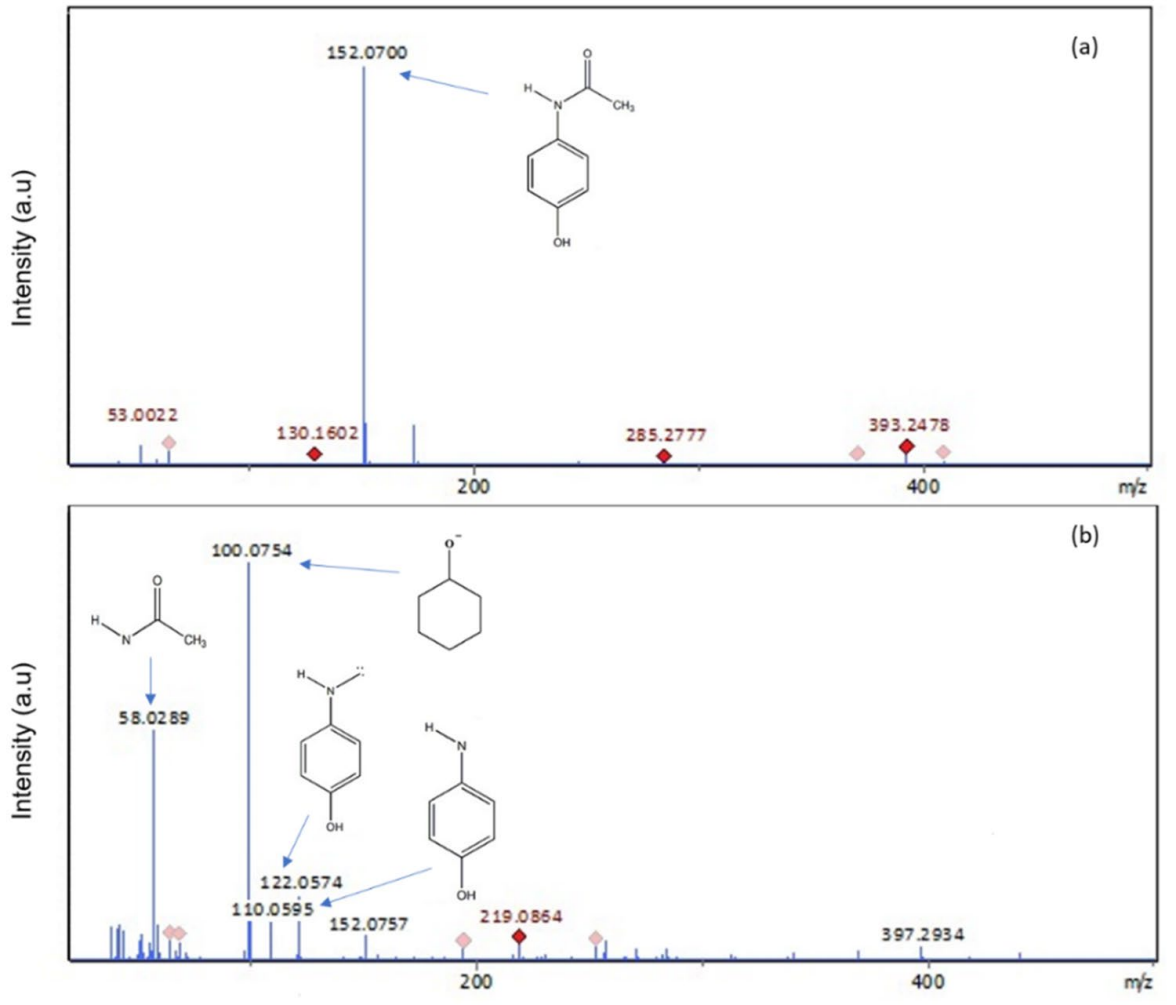
Mass spectra of paracetamol intermediates (**a**) before degradation and (**b**) after degradation with ZnO/g-C_3_N_4_10% composite.

The identification of intermediates generated by the degradation of paracetamol was done by LC-QTOF-MS analysis. The outcomes depicted in Fig. 12a provide evident molecular ion peak at m/z = 152 referred to paracetamol^82^. However, after degradation (Fig. 12b), the hydroxyl radical (^•^OH) favored the braking of CH_3_–C=O in paracetamol molecule to formulate hydroquinone (m/z = 110) which further transformed into cyclohexanol (m/z = 100) by the attack of ^•^OH on the amide group^83^. Phenethyl alcohol (m/z = 122) and another element at m/z = 58 are also among the identified species, moreover, a series of reactions among the formed compounds could produce further more compounds and eventually form water and CO_2_^84^.

## Conclusion

In this study, a practicable and efficient visible light sensitive photocatalytic composite, ZnO/g-C_3_N_4_, was synthesized successfully by hydrothermal and calcination methods. Four different compositions were formulated by incorporating g-C_3_N_4_ into ZnO at varying concentrations among which the optimized photocatalyst, ZnO/g-C_3_N_4_10%, exhibited the best activity for the degradation of paracetamol in aqueous solution. The addition of g-C_3_N_4_ into ZnO matrix has led to a reduction in the band gap from 3.13 to 2.39 eV which resulted in a significant enhancement in light absorption and charge carrier separation. Moreover, a noteworthy increase in the S_BET_ from 2.33 to 11.33 m^2^/g and enhanced morphology provided the grounds for higher activity of the photocatalyst. The study revealed that hydroxyl radicals played a pivotal role in photooxidation and a proposed pathway for degradation was postulated. The intrinsic merits of the synthesized composite such as its ability to actively respond in visible light, straightforward and cost-effective preparation followed by its evident effectiveness makes it a promising catalyst for prospective applications.

## Data Availability

All data generated or analyzed during this study are included in this published article. All the data are stored on the server of Sharjah University and can be provided upon request from the corresponding author (fahad.hassan@sharjah.ac.ae).
